# Supplementary material for: HIVprotI: an integrated web based platform for prediction and design of HIV proteins inhibitors
Source: J Cheminform. 2018 Mar 9;10:12. doi: 10.1186/s13321-018-0266-y (PMC5845081; doi:10.1186/s13321-018-0266-y)
Supplement: Supplementary file 2 — Additional file 2. Source code of HIVProtI web server. [file 13321_2018_266_MOESM2_ESM.zip › HIVprotI_Source-code/hivprotI/weka-3-6-6/doc/weka/gui/beans/BeanConnection.html]

BeanConnection


---


|  |  |  |
| --- | --- | --- |
| **Overview** \| **Package** \| **Class** \| **Tree** \| **Deprecated** \| **Index** \| **Help** \| **Weka's home** | |  |
| **PREV CLASS**   **NEXT CLASS** | **FRAMES**    **NO FRAMES**     **All Classes** |  |
| SUMMARY: NESTED \| FIELD \| CONSTR \| METHOD | DETAIL: FIELD \| CONSTR \| METHOD |  |


---


## weka.gui.beans Class BeanConnection

```
java.lang.Object
  weka.gui.beans.BeanConnection
```

**All Implemented Interfaces:**: java.io.Serializable

---

``` public class BeanConnection extends java.lang.Object implements java.io.Serializable ```

Class for encapsulating a connection between two beans. Also
maintains a list of all connections

**Version:**
:   $Revision: 7059 $

**Author:**
:   Mark Hall

**See Also:**: Serialized Form

---

| **Field Summary** | |
| --- | --- |
| `static java.util.Vector` | `CONNECTIONS`             The list of connections |


**Constructor Summary**

`BeanConnection(BeanInstance source,
BeanInstance target,
java.beans.EventSetDescriptor esd)`
  
          Creates a new `BeanConnection` instance.


| **Method Summary** | |
| --- | --- |
| `static java.util.Vector` | `associatedConnections(java.util.Vector subFlow)`             Returns a vector of BeanConnections associated with the supplied vector of BeanInstances, i.e. |
| `static void` | `doMetaConnection(BeanInstance source, BeanInstance target, java.beans.EventSetDescriptor esd, javax.swing.JComponent displayComponent)` |
| `static java.util.Vector` | `getClosestConnections(java.awt.Point pt, int delta)`             Return a list of connections within some delta of a point |
| `static java.util.Vector` | `getConnections()`             Returns the list of connections |
| `java.lang.String` | `getEventName()`             Returns the name of the event for this conncetion |
| `BeanInstance` | `getSource()`             returns the source BeanInstance for this connection |
| `BeanInstance` | `getTarget()`             Returns the target BeanInstance for this connection |
| `static java.util.Vector` | `inputs(java.util.Vector subset)`             Returns a vector of BeanInstances that can be considered as inputs (or the left-hand side of a sub-flow) |
| `boolean` | `isHidden()`             Returns true if this connection is invisible |
| `static java.util.Vector` | `outputs(java.util.Vector subset)`             Returns a vector of BeanInstances that can be considered as outputs (or the right-hand side of a sub-flow) |
| `static void` | `paintConnections(java.awt.Graphics gx)`             Renders the connections and their names on the supplied graphics context |
| `void` | `remove()`             Remove this connection |
| `static void` | `removeConnections(BeanInstance instance)`             Remove all connections for a bean. |
| `static void` | `reset()`             Reset the list of connections |
| `static void` | `setConnections(java.util.Vector connections)`             Describe `setConnections` method here. |
| `void` | `setHidden(boolean hidden)`             Make this connection invisible on the display |

**Methods inherited from class java.lang.Object**

`equals, getClass, hashCode, notify, notifyAll, toString, wait, wait, wait`

**Field Detail**

### CONNECTIONS

```
public static java.util.Vector CONNECTIONS
```

:   The list of connections


**Constructor Detail**

### BeanConnection

```
public BeanConnection(BeanInstance source,
                      BeanInstance target,
                      java.beans.EventSetDescriptor esd)
```

:   Creates a new `BeanConnection` instance.

    **Parameters:**: `source` - the source bean: `target` - the target bean: `esd` - the EventSetDescriptor for the connection be displayed


**Method Detail**

### reset

```
public static void reset()
```

:   Reset the list of connections

---


### getConnections

```
public static java.util.Vector getConnections()
```

:   Returns the list of connections

    :   **Returns:**: the list of connections

---


### setConnections

```
public static void setConnections(java.util.Vector connections)
```

:   Describe `setConnections` method here.

    :   **Parameters:**: `connections` - a `Vector` value

---


### associatedConnections

```
public static java.util.Vector associatedConnections(java.util.Vector subFlow)
```

:   Returns a vector of BeanConnections associated with
    the supplied vector of BeanInstances, i.e. all connections
    that exist between those BeanInstances in the subFlow.

    :   **Parameters:**: `subFlow` - a Vector of BeanInstances **Returns:**: a Vector of BeanConnections

---


### inputs

```
public static java.util.Vector inputs(java.util.Vector subset)
```

:   Returns a vector of BeanInstances that can be considered
    as inputs (or the left-hand side of a sub-flow)

    :   **Parameters:**: `subset` - the sub-flow to examine **Returns:**: a Vector of inputs to the sub-flow

---


### outputs

```
public static java.util.Vector outputs(java.util.Vector subset)
```

:   Returns a vector of BeanInstances that can be considered
    as outputs (or the right-hand side of a sub-flow)

    :   **Parameters:**: `subset` - the sub-flow to examine **Returns:**: a Vector of outputs of the sub-flow

---


### paintConnections

```
public static void paintConnections(java.awt.Graphics gx)
```

:   Renders the connections and their names on the supplied graphics
    context

    :   **Parameters:**: `gx` - a `Graphics` value

---


### getClosestConnections

```
public static java.util.Vector getClosestConnections(java.awt.Point pt,
                                                     int delta)
```

:   Return a list of connections within some delta of a point

    :   **Parameters:**: `pt` - the point at which to look for connections: `delta` - connections have to be within this delta of the point **Returns:**: a list of connections

---


### removeConnections

```
public static void removeConnections(BeanInstance instance)
```

:   Remove all connections for a bean. If the bean is a target for
    receiving events then it gets deregistered from the corresonding
    source bean. If the bean is a source of events then all targets
    implementing BeanCommon are notified via their
    disconnectionNotification methods that the source (and hence the
    connection) is going away.

    :   **Parameters:**: `instance` - the bean to remove connections to/from

---


### doMetaConnection

```
public static void doMetaConnection(BeanInstance source,
                                    BeanInstance target,
                                    java.beans.EventSetDescriptor esd,
                                    javax.swing.JComponent displayComponent)
```

---


### setHidden

```
public void setHidden(boolean hidden)
```

:   Make this connection invisible on the display

    :   **Parameters:**: `hidden` - true to make the connection invisible

---


### isHidden

```
public boolean isHidden()
```

:   Returns true if this connection is invisible

    :   **Returns:**: true if connection is invisible

---


### remove

```
public void remove()
```

:   Remove this connection

---


### getSource

```
public BeanInstance getSource()
```

:   returns the source BeanInstance for this connection

    :   **Returns:**: a `BeanInstance` value

---


### getTarget

```
public BeanInstance getTarget()
```

:   Returns the target BeanInstance for this connection

    :   **Returns:**: a `BeanInstance` value

---


### getEventName

```
public java.lang.String getEventName()
```

:   Returns the name of the event for this conncetion

    :   **Returns:**: the name of the event for this connection


---


|  |  |  |
| --- | --- | --- |
| **Overview** \| **Package** \| **Class** \| **Tree** \| **Deprecated** \| **Index** \| **Help** \| **Weka's home** | |  |
| **PREV CLASS**   **NEXT CLASS** | **FRAMES**    **NO FRAMES**     **All Classes** |  |
| SUMMARY: NESTED \| FIELD \| CONSTR \| METHOD | DETAIL: FIELD \| CONSTR \| METHOD |  |


---
